# Neonatal overfeeding attenuates microgliosis and hippocampal damage in an infant rat model of pneumococcal meningitis

**DOI:** 10.3389/fimmu.2024.1429157

**Published:** 2024-10-14

**Authors:** Larissa Marcely Gomes Cassiano, Karina Barbosa de Queiroz, Thais Veronez de Andrade Martins, Ana Luiza Azevedo Maia, Milene Alvarenga Rachid, Sarah J. Spencer, Roney Santos Coimbra

**Affiliations:** ^1^ Neurogenômica, Imunopatologia, Instituto René Rachou (IRR), Fiocruz, Belo Horizonte, Brazil; ^2^ Departamento de Bioquímica e Imunologia, Instituto de Ciências Biológicas, Universidade Federal de Minas Gerais (UFMG), Belo Horizonte, Brazil; ^3^ Serviço de Animais de Laboratório (SAL), Instituto Nacional de Controle de Qualidade em Saúde (INCQS), Fiocruz, Rio de Janeiro, Brazil; ^4^ Departamento de Patologia Geral, Instituto de Ciências Biológicas, Universidade Federal de Minas Gerais (UFMG), Belo Horizonte, Brazil; ^5^ School of Health Sciences and Biomedical Sciences, Royal Melbourne Institute of Technology (RMIT) University, Melbourne, VIC, Australia

**Keywords:** pneumococcal meningitis, neonatal overfeeding, hippocampus, neuroinflammation, microglia

## Abstract

**Background:**

Pneumococcal meningitis (PM) triggers apoptotic neuronal and progenitor cell death in the hippocampal dentate gyrus (DG), resulting in subsequent cognitive impairment. Microglia play a crucial role in PM-induced hippocampal damage. While the lasting effects of neonatal nutrition on health are well documented, the influence of early-life overfeeding on the host response to neuroinfections remains uncertain. This study aimed to examine whether neonatal overfeeding affects the outcome of PM in the hippocampus (HC).

**Material and methods:**

Overfeeding was induced by adjusting litter size immediately after birth. On the eleventh day of life, rats were intracisternally injected with *Streptococcus pneumoniae* or saline, followed by euthanasia after 24 hours for brain dissection. Histological analysis evaluated apoptosis in the DG and the extent of inflammatory infiltrate in the hippocampal fissure, while microgliosis was assessed by immunohistochemistry. The hippocampal transcriptome was analyzed using RNAseq, and the mRNA levels of specific inflammatory biomarkers were evaluated via RT-qPCR.

**Results:**

Overfed rats exhibited 40.5% greater body mass compared to their normal-fed counterparts. Intriguingly, PM-induced apoptosis in the DG was 50% lower in overfed rats. This effect was accompanied by significant alterations in the hippocampal transcriptional profile, particularly the lack of activation of the *Programmed cell death* pathway in overfed infected animals. RT-qPCR analysis of *Aif1* and examination of Iba1-immunostained cells revealed mild microgliosis in the HC of infected-overfed animals. This reduced microglial reaction may be attributed to the diminished activation of interferon signaling pathways, as disclosed by the transcriptome analysis, potentially preventing microglial priming. Additionally, evidence of reduced neuroinflammation in overfed rats with PM was observed through the milder activation of pathways associated with Toll-like receptors, interleukins, and chemokine signaling. Furthermore, overfed animals exhibited increased transcription of proinflammatory *Il6* and anti-inflammatory *Il10* genes, with the latter showing higher expression even in the absence of PM, suggesting a priming effect of overfeeding on hippocampal immune cells.

**Conclusion:**

This study sheds light on the complex interplay between early-life overfeeding, immune response, and neuroprotection in infant rats with PM. The findings demonstrate the neuroprotective impact of early-life overfeeding in the context of PM, linked to the modulation of microglial function.

## 1 Introduction

Pneumococcal meningitis (PM) is a severe infection of central nervous system (CNS) caused by *Streptococcus pneumoniae* and is characterized by an intense inflammatory response in the meninges and cerebral ventricles. Pneumococcus is a leading cause of bacterial meningitis, and it associated with the highest mortality and morbidity rates among children and the elderly (1, 2). The increased severity and lethality of PM result from an intricate interplay of host response factors and bacterial pathogenicity elements [reviewed in (3):]. Notably, patients with PM exhibit elevated levels of Interferon-gamma (IFN-γ), Chemokine (C-C motif) ligand 2 (CCL2), and Matrix Metalloproteinase 9 (MMP-9) in their cerebrospinal fluid (CSF) compared to those with other aetiologies of bacterial meningitis, indicating a more intense inflammatory response (4). IFN-γ stands out as a pivotal regulator of this process, given its role in activating macrophages and microglia, and its collaboration with interleukin-1 beta (IL-1β) to regulate the production of other cytokines (5–8).

Microglia-mediated inflammation has been recognized as the primary mediator of the neuronal dysfunction associated with PM [reviewed in (9, 10):]. During the inflammatory process induced by PM in the CNS, microglia release pro-inflammatory factors and reactive species that promote oxidative stress, ultimately leading to neuronal death, particularly in the cortex and hippocampus (HC) (11–14). Within the hippocampal dentate gyrus (DG), the infection triggers apoptosis in postmitotic neurons and progenitor cells, thereby contributing to the significant cognitive deficits observed in survivors of PM (13, 15, 16).

Microglia are highly sensitive to environmental programming effects during the neonatal period, with dietary factors acknowledged as significant contributors to this early sensitization [reviewed in (17):]. Previous studies indicate that neonatal overfeeding, associated with an elevated microglial density in the HC, is correlated with long-term cognitive dysfunction (18, 19). However, the interaction between neonatal overfeeding and the host response to PM has not yet been investigated. In this context, the present study explores the impact of neonatal overfeeding on the hippocampal inflammatory response to PM in infant rats.

## 2 Materials and methods

### 2.1 Animal model and experimental design

All the experimental procedures were approved by the Ethics Committee of Care and Use of Laboratory Animals (CEUA-FIOCRUZ, protocol LW-22/13) and were conducted in accordance with the regulations described in the Committee’s Guiding Principles Manual.

A previously published model of neonatal overfeeding was used (18). Briefly, this litter size manipulation model consists of distributing Wistar rat pups immediately after birth into two types of litters: those with 10 pups per dam (normal feeding) and those with four pups per dam (overfeeding). At postnatal day 11th, the normal and overfed litters were further subdivided into two groups: a) animals infected by intracisternal injection of 10 μL solution containing ~3 × 10^6^ cfu/mL of *S. pneumoniae* (serotype 3, strain 38/12 MEN provided by Fundação Ezequiel Dias) in saline; b) animals sham-infected with an intracisternal injection of 10 μL of sterile saline, as previously described (20).

Eighteen hours post infection (p.i.) all rats were weighed and clinically scored by the following score system: 1 for comatose animals, 2 for rats that do not turn upright after positioning on the back, 3 for animals that turn within 30 seconds, 4 for animals that turn within less than 5 seconds, and 5 for rats with normal activity. Infection was documented by quantitative culture of 10 μL of CSF, and all animals received antibiotic therapy (100 mg/kg of body mass of ceftriaxone, EMS Sigma Pharma Ltda., Hortolândia, Brazil, subcutaneously) before returned to their dams. Antibiotic treatment was administered to reduce animal suffering and ensure their survival throughout the experimental period. Twenty-four hours p.i., rats were euthanized by intraperitoneal overdose of Ketamine (300 mg/kg) + Xylazine (30 mg/kg) (Syntec, Hortolândia, Brazil).

Immediately after euthanasia, animals were perfused via the left cardiac ventricle with 7.5 mL of RNAse-free ice-cold phosphate buffered saline (PBS). The brains were removed from the skulls and dissected. The right hemisphere was postfixed in 4% paraformaldehyde (PFA) (Sigma, São Paulo, Brazil) and further processed for histopathological assessment. The HC from the left hemisphere was excised and preserved in RNA later at 4°C for 24 hours before being stored at -80°C until further use.

Only animals with a bacterial titer of at least 10_8_ cfu/mL in the CSF at 18 hours p.i. were included in this study.

### 2.2 Hippocampal histopathological analysis

The right hemispheres of the brains were embedded in paraffin and then sectioned into 5 µm-thick coronal sections using a Leica CM1850 microtome. (Leica, Wetzlar, Germany).

To quantify apoptotic cells in the lower blade of the hippocampal DG granular layer, sections stained with Cresyl violet were examined under optical microscopy with a 40× objective. Neurons exhibiting morphological characteristics indicative of apoptosis, such as condensed or fragmented nuclei and apoptotic bodies, were counted in three distinct visual fields of the DG lower blade for each animal.

The inflammatory infiltrate into the hippocampal fissure was estimated with a semiquantitative method described by Tauber et al. (21). Sections were stained with hematoxylin and eosin (H&E) and visualized using one high-power field (20× objective). The granulocyte invasion was scored using the following score system: No granulocytes = 0; < 10 granulocytes = 1; 10–50 granulocytes = 2; and > 50 granulocytes: = 3.

To assess microgliosis and microglia activation, sections were immunostained using the Novolink Polymer Detection System (Leica Biosystems, Nussloch, Germany), following the manufacturer’s instructions. The process included the use of a primary rabbit monoclonal antibody against rat Ionized calcium-binding adaptor molecule 1 (Iba1) (Abcam, Cambridge, UK; <ns/>AB178847) at a dilution of 1:8000. Sections were counterstained with hematoxylin. Aperio Scanner (Leica Biosystems) and the software Aperio ImageScope version 12.4.6 (Leica Biosystems) and Fiji (Image J) (22) were used to capture and analyze images of the histological sections, respectively. Four distinct fields (40× objective, 20× eye piece) of the DG granular layer of each rat were analyzed. Iba1-positive cells were quantified, and the Skeleton Analysis method, developed by Young et al. (23), was employed to assess changes in microglial morphology as an indicator of microgliosis. Data were represented by normalizing the endpoints (cell branch tips) in relation to branch lengths, where the highest values represent the less reactive microglial state.

### 2.3 Total RNA preparation

The hippocampal samples were dissociated, and the cells were lysed using the TissueLyser LT (Qiagen, Hilden, Germany) with a combination of Trizol reagent (Thermo Fischer, Waltham, MA) and chloroform (Merck Millipore, Burlington, MA) for RNA extraction. Then, total RNA was purified using the miRNeasy Mini Kit (Qiagen) according to the manufacturer’s protocol. Total RNA was treated with RNase-Free DNase Set (Qiagen), following to the manufacturer’s instructions. Total RNA was quantified using the Qubit 2.0 Fluorometer (Life Technologies, Carlsbad, CA) and RNA integrity was analyzed by Agilent RNA 6000 Nano (Agilent Technologies, Waldbronn, Germany).

### 2.4 RNA-seq

Three RNA samples from each group (Infected normal-fed, Infected overfed, Sham-infected normal-fed and Sham-infected overfed) were used to produce cDNA libraries with the TruSeq Stranded mRNA Kit (Illumina, San Diego, CA). The indexed libraries were sequenced using the TG NextSeq 500/550 High Output Kit v2 (Illumina).

The sequenced raw reads were processed with Trimmomatic (24) to remove low-quality bases and reads less than 36 nt. Reads were subsequently aligned to *Rattus norvegicus* reference genome (release 104) using the software STAR (25). Uniquely mapped reads were log_2_-normalized with the *rlog* function from the DESeq2 R package (26).

The study analyzed the impact of overfeeding (comparing Sham-infected normal-fed to Sham-infected overfed) and meningitis (comparing Infected normal-fed to Sham-infected normal-fed, and Infected overfed to Sham-infected overfed) on canonical pathways from the Reactome database. This evaluation was conducted using Gene Set Enrichment Analysis (GSEA) (27) software with the following settings: Permutations type: gene set; Number of permutations: 1000; Metric for ranking genes: tTest; Gene set cut-off: FDR < 0.05; Other parameters: default.

### 2.5 RT-qPCR for analysis of Aif-1 and selected inflammation-related genes

Two hundred nanograms of total RNA were reverse transcribed into cDNA using the High-Capacity cDNA RT kit (Thermo Fischer). mRNA expression was quantified by real-time quantitative PCR using the Taqman system (Thermo Fischer), and the ABI 7500 Fast Real-Time PCR System was used to detect the target. Rat-specific primers/probes were used to detect *Aif1* (Allograft Inflammatory Factor 1) (Rn00574125_g1), *Il1b* (Interleukin-1 beta) (Rn00580432_m1), *Il6* (Interleukin-6) (Rn014110330_m1), *Il10* (Interleukin-10) (Rn01483988_g1), *Tnf* (Tumor Necrosis Factor) (Rn01525859_g1), and *Ccl2* (C-C motif chemokine ligand 2) (Rn00580555_m1). Ct data were normalized by *Rplp2* (Ribosomal Protein, Large, P2) (Rn01479927_g1) expression, which was stably expressed in all experimental groups. The mean ΔCt from sham-infected normal-fed group was utilized as calibrator to calculate the relative gene expression using the 2^−ΔΔCt^ method (28).

### 2.6 Statistical analysis

The statistical analyses were performed using Graph Pad Prism 8 (GraphPad Software Inc., Irvine, CA) (version 8.0.2). The Shapiro–Wilk test was used to verify the normal distribution of data and statistical tests were chosen according to the experimental design. Two-tailed Mann-Whitney test was used to compare two groups with non-parametric distribution and Two-way ANOVA followed by Bonferroni or Tukey’s test was used when comparisons involved the effect of two factors on a dependent variable in three or more groups. Data were expressed as median ± interquartile range or mean ± standard deviation. Differences were considered significant when *P*-values were smaller than 0.05.

## 3 Results

### 3.1 Neonatal overfeeding and pneumococcal meningitis models

**Figure 1:**
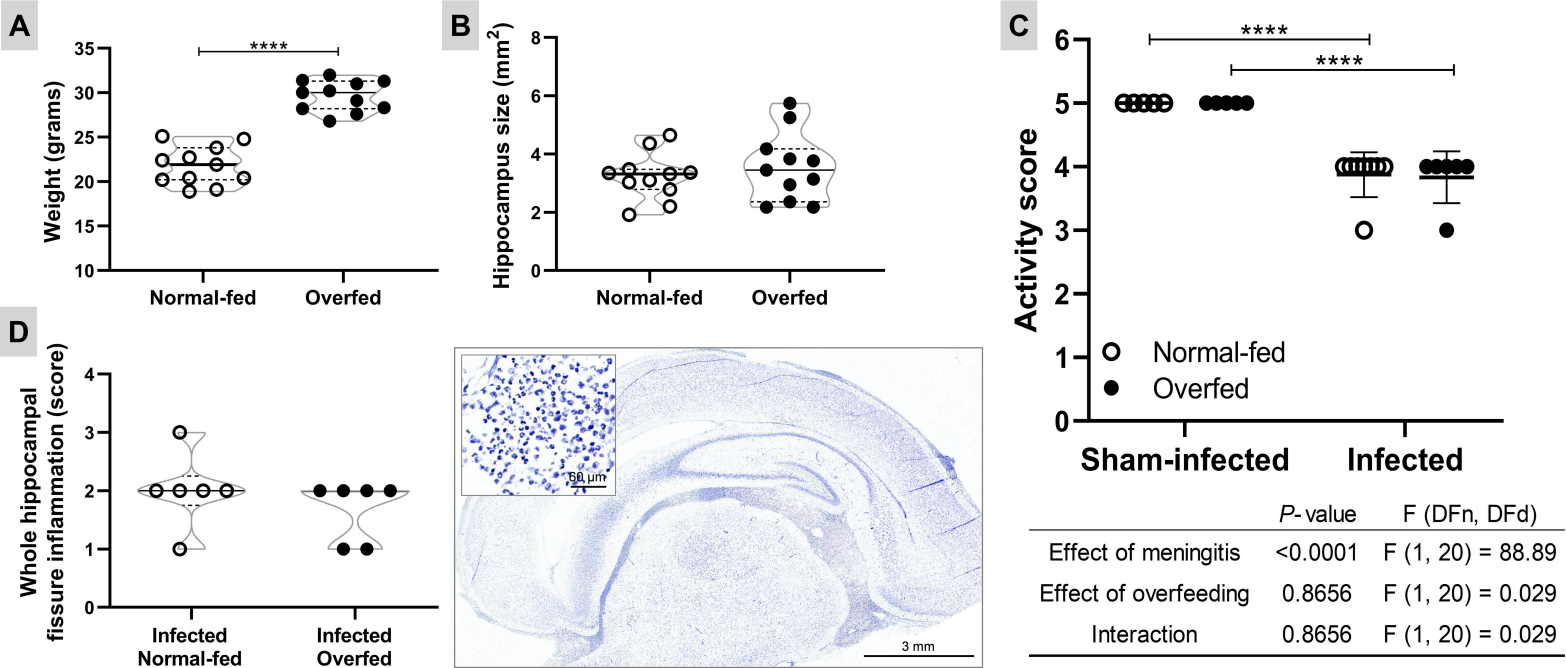
Neonatal overfeeding and pneumococcal meningitis models Body weight at the 11th day after birth **(A)** and hippocampus size **(B)**. The effect of the neonatal overfeeding was evaluated using the two-tailed unpaired T test. Horizontal bars represent median (bold) and quartiles (dashed). **** *P* < 0.0001. **(C)** Activity score. The animals were clinically scored by the following score system at 18 hours post infection: 1 for comatose animals, 2 for rats that do not turn upright after positioning on the back, 3 for animals that turn within 30 seconds, 4 for animals that turn within less than 5 seconds, and 5 for rats with normal activity. The effects of the meningitis and the neonatal overfeeding in the activity score were compared using a two-way ANOVA followed by Tukey’s multiple comparisons test. Horizontal bars represent mean ± standard deviation. **** *P* < 0.0001. **(D)** Inflammatory infiltrate in the hippocampal fissure. Left: The effect of the infection in normal and overfed animals was assessed using the two-tailed Mann-Whitney test. Horizontal bars represent median (bold) and quartiles (dashed). Right: Histological section showing the inflammatory infiltrate in hippocampal fissure (1×) of an infected animal treated with placebo. In detail, a zoom (40×) of the polymorphonuclear leukocytes present in the hippocampal fissure.

Overfeeding was effectively induced by adjusting the litter size (four pups per dam for overfeeding or 10 pups per dam for normal feeding). By the 11th day after birth, the overfed animals exhibited a 40.5% increase in body mass compared to the normal-fed (*P* < 0.0001, t = 9.345, df = 20), with no significant changes in hippocampal sizes (*P* = 0.470, t = 0.735, df = 20) (**Figure 1**). The bacterial titre in the CSF at 18 hours p.i. showed no significant difference between overfed and normal-fed animals (means 1.91 x 10^8^ *vs*. 1.70 x 10^8^, *P* = 0.364, t = 0.945, df = 12), confirming comparable infection intensities in both groups. Meningitis decreased the activity score of the infant rats compared to sham-infected controls (4 vs. 5, *P* < 0.001), while overfeeding did not impact this parameter (**Figure 1**). Moreover, overfeeding had no impact on the intensity of the inflammatory infiltrate in the HC fissure during PM (*P* = 0.636, U = 13) (**Figure 1**).

### 3.2 Neonatal overfeeding attenuates apoptosis in infant rats with pneumococcal meningitis

**Figure 2:**
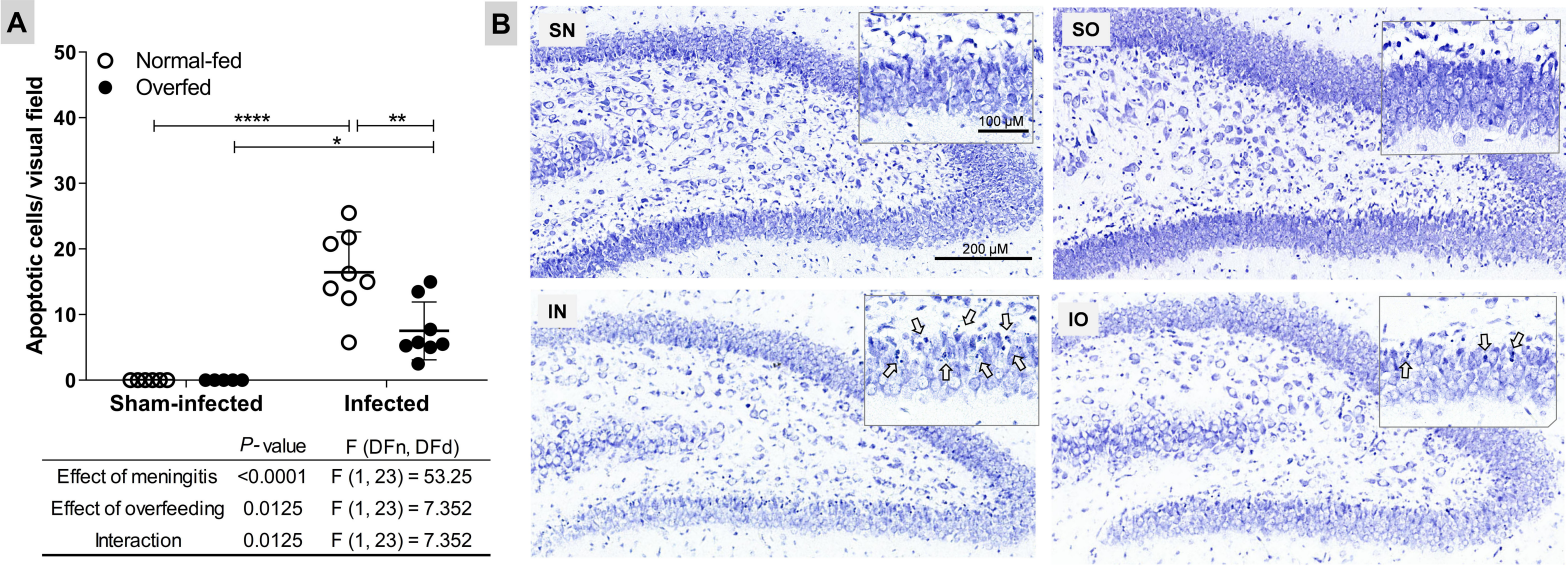
Neonatal overfeeding attenuates apoptosis in the dentate gyrus granular layer of infant rats with pneumococcal meningitis **(A)** Apoptotic cells. The effects of meningitis and neonatal overfeeding were evaluated using a two-way ANOVA followed by Tukey’s multiple comparisons tests. Horizontal bars represent the mean ± standard deviation. *P < 0.05; **P < 0.01; ***P < 0.001. **(B)** Nissl-stained histological sections showing the hippocampal dentate gyrus (6×). The details show amplified sections of the granular layer on the lower blade of the dentate gyrus (20×), arrows points to apoptotic cells. SN, Sham-infected normal-fed; SO, Sham-infected overfed; IN, Infected normal-fed; IO, Infected overfed.

The absolute number of apoptotic cells in the DG granular layer is depicted in **Figure 2**. As expected, PM significantly increased apoptosis (*P* < 0.0001) in normal-fed animals. In overfed infected infant rats, there was a 50% reduction in apoptotic cells in the hippocampal DG compared to normal-fed infected animals.

### 3.3 Neonatal overfeeding mitigates PM-induced microglia activation but did not affect the intensity of the inflammatory infiltrate

**Figure 3:**
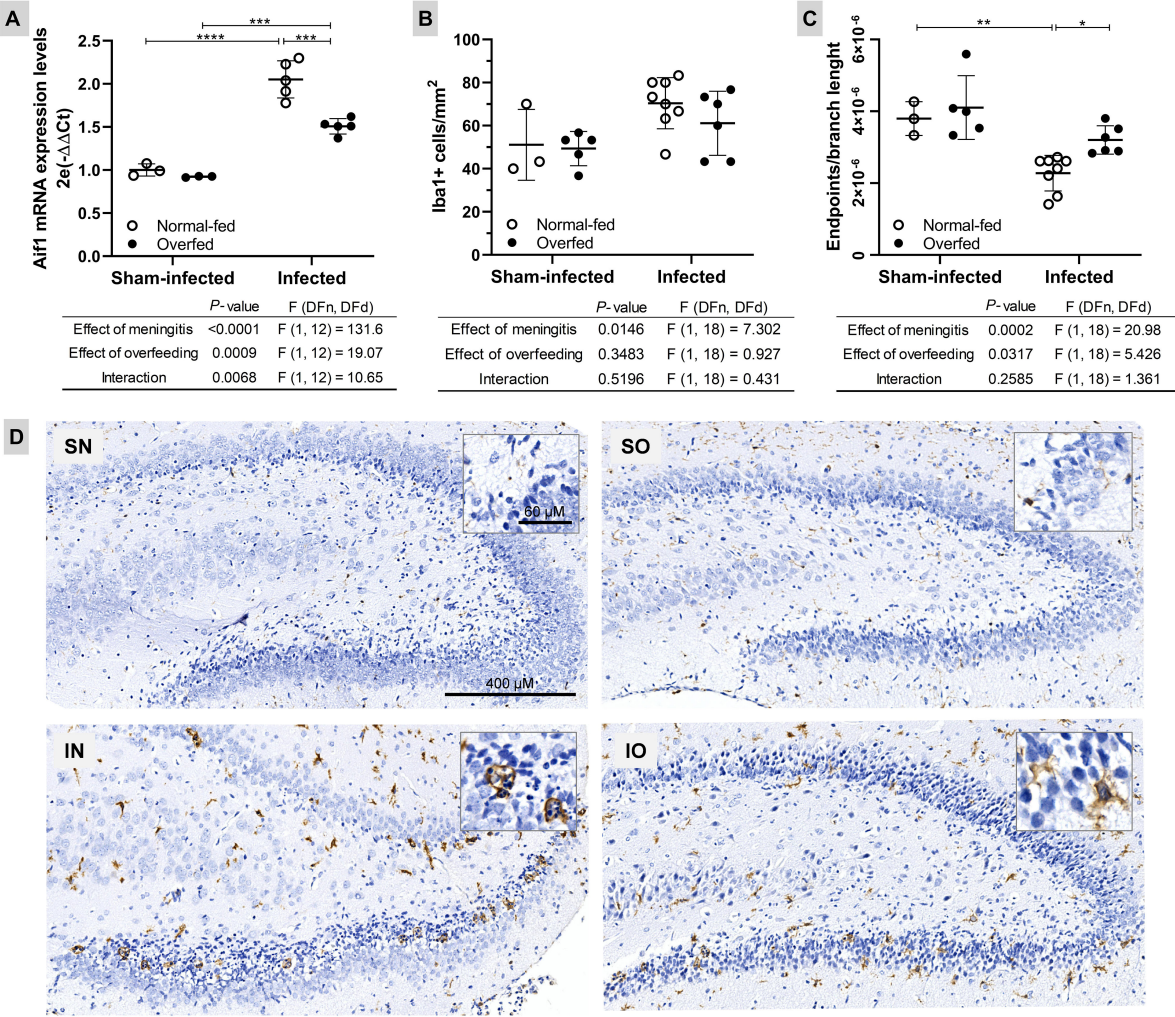
Neonatal overfeeding attenuates microglia activation in the dentate gyrus of infant rats with pneumococcal meningitis **(A)** The *Aif1* mRNA expression was evaluated using the 2^−ΔΔCt^ method, with the mean expression levels from the sham-infected normal-fed group serving as the calibrator. *Rplp2* was used as a reference gene. **(B)** Microglia count (Iba1+ cells/mm^2^) in the hippocampus dentate gyrus lower blade. **(C)** Microglia morphology (Iba1+ cells endpoints\branch length). The highest values correspond to a less reactive microglial state (cells with branched morphology and smaller size). For all plots, the effects of meningitis and neonatal overfeeding were compared using two-way ANOVA followed by Tukey’s multiple comparisons tests. Horizontal bars represent the mean ± standard deviation. *P < 0.05; **P < 0.01; ***P < 0.001; ****P < 0.0001. **(D)** Hippocampal sections (6×) from both normal-fed (left) and overfed (right) animals, whether sham-infected or infected, were subjected to immunostaining for Iba1. In detail, zooms (40×) of typical ameboid (more reactive/pro-inflammatory) and intermediate (less reactive) microglial cells, respectively. SN, Sham-infected normal-fed; SO, Sham-infected overfed; IN, Infected normal-fed; IO, Infected overfed.

To explore the impact of neonatal overfeeding on the microglial response to PM, the mRNA levels of the biomarker gene *Aif1* were assessed in the HC using RT-qPCR (**Figure 3**). The results revealed that PM up-regulates *Aif1* in both normal-fed (*P* < 0.0001) and overfed infected (*P* < 0.001) rats compared to their respective sham-infected controls (**Figure 3A**). However, overfed infected animals exhibited lower *Aif1* expression (*P* < 0.001) than normal-fed animals. This finding is supported by the results of the morphological analysis of microglia in the DG granular layer (**Figures 3B, D**), where the highest values correspond to the lowest microglial complexity (cells with a more branched morphology and a smaller circumference, as detailed in **Figure 3D**. There was no statistically significant difference between the groups regarding the number of Iba1+ cells/mm^2^ in the hippocampal DG (**Figure 3B**). Thus, neonatal overfeeding attenuates the pro-inflammatory profile of this local immune cell population during PM.

### 3.4 The anti-inflammatory and neuroprotective impact of neonatal overfeeding on the host response to PM is associated with alterations in the hippocampal transcriptional profile

**Figure 4:**
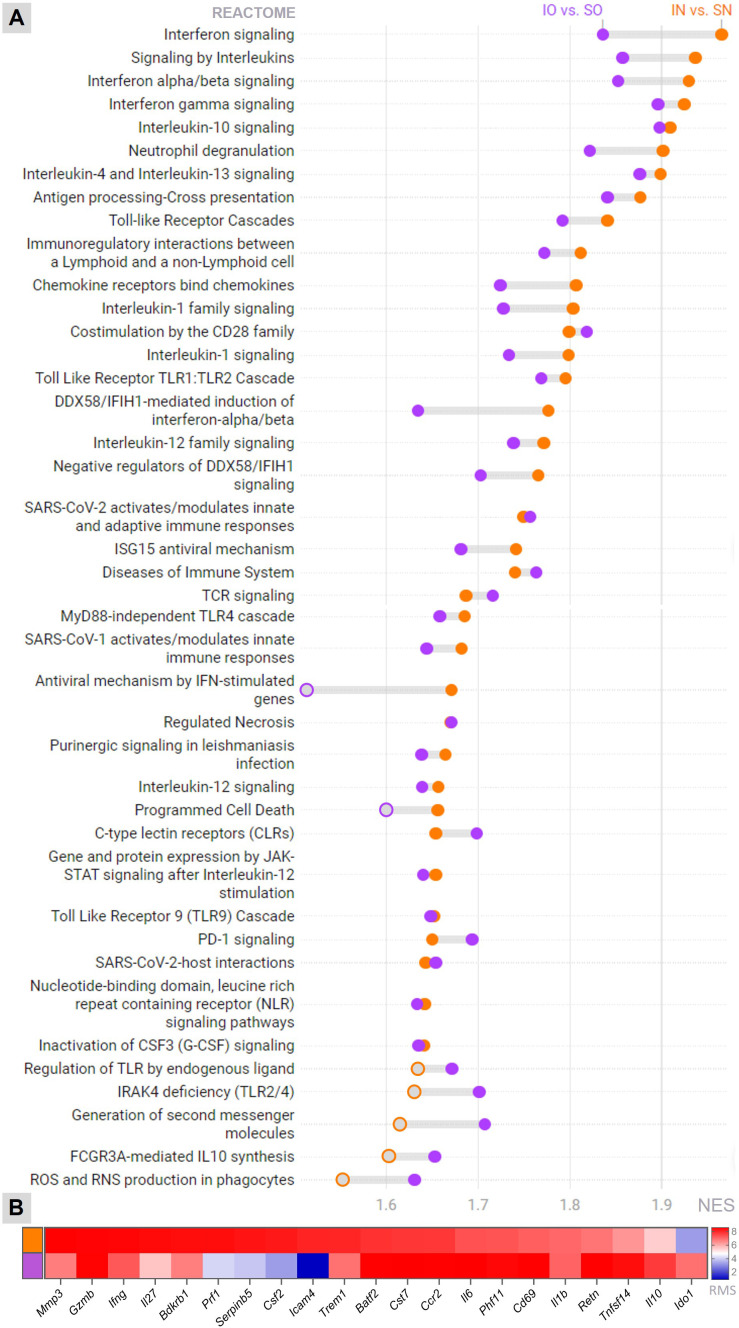
Transcriptome analysis Canonical pathways (Reactome) **(A)** and the top biomarkers **(B)** affected by meningitis in the hippocampus of infant rats, whether overfed or normal-fed, were identified through functional enrichment analysis. The threshold for statistical significance in pathway’s enrichment was set at an adjusted *P*-value (FDR) < 0.01. IN, Infected normal-fed; IO, Infected overfed; SN, Sham-infected normal-fed; SO, Sham-infected overfed; NES, Normalized Enrichment Score; RMS, Rank Metric Score Orange represents the Infected normal-fed *versus* Sham-infected normal-fed contrast, and purple represents the Infected overfed *versus* Sham-infected overfed contrast. Filled orange or purple circles = *P*-value (FDR) < 0.01.

To unveil the molecular mechanisms underlying the anti-inflammatory and neuroprotective effects of neonatal overfeeding in infant rats with PM, as evidenced by phenotypic analysis, the hippocampal transcriptional profiles were compared among the four experimental groups. As shown in **Figure 4A**, several canonical pathways pertinent to the central processes in pathophysiology of PM (14, 29) are enriched with differentially expressed genes (DEG) in both normal-fed and overfed infected animals compared to their respective sham-infected control groups. However, for most of these pathways, the GSEA’s Normalized Enrichment Score (NES) is smaller in the contrast between infected overfed and sham-infected overfed groups than in the contrast between infected normal-fed and sham-infected normal-fed groups. These pathways encompass Toll-like receptor cascades, interferon, interleukins, and chemokines signaling, as well as Neutrophil degranulation. Nevertheless, the canonical pathways related to the antiviral mechanisms of IFN-stimulated genes and programmed cell death did not show significant enrichment in DEG during PM among the overfed rats. Furthermore, a few pathways, including *Regulation of TLR by endogenous ligands*, *FCGR3A-mediated IL10 synthesis*, and *Production of ROS and RNS in phagocytes* were exclusively enriched in the comparison between overfed infected and overfed sham-infected animals. No canonical pathway from the Reactome database was enriched in the DEG when comparing the sham-infected overfed group with the sham-infected normal-fed groups. The functional enrichment analysis also disclosed biomarker genes associated to the host response to PM in overfed or normal-fed infant rats (**Figure 4B**). Notably, pivotal genes in the pathophysiology of PM showed different expression patterns depending on the feeding regimen. Overfed infected animals displayed a dampened expression of pro-inflammatory biomarkers, such as *Mmp3*, *Infg*, *Il27*, and *Trem1*, alongside a notable increase in the anti-inflammatory *Il10*. Additional findings from the hippocampal transcriptome analysis are presented in **Supplementary Tables 1**, **2**.

### 3.5 *Il6* and *Il10* are likely central mediators influencing the effects of neonatal overfeeding on the HC

**Figure 5:**
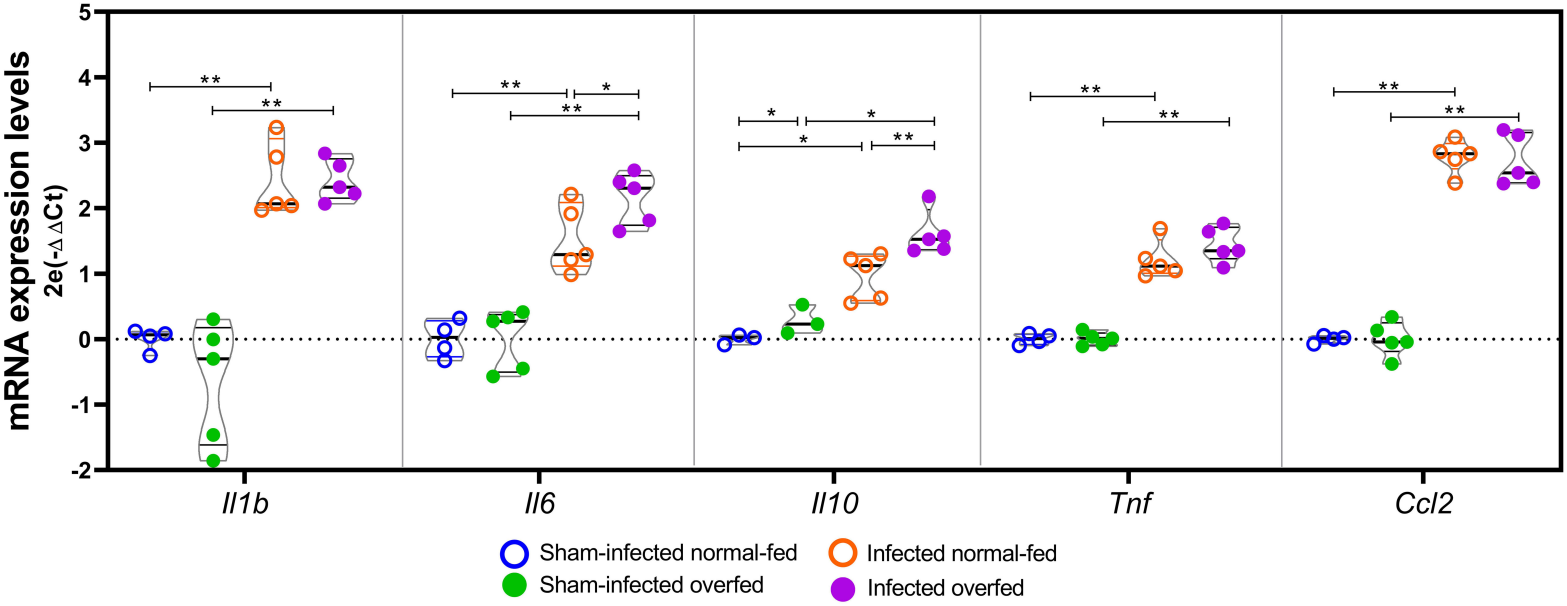
RT-qPCR analysis of neuroinflammation biomarkers The *Il1b, Il6, Il10, Tnf, and Ccl2* mRNA levels were assessed using the 2^-Ct^ method, with the mean expression levels from the sham-infected normal-fed group serving as the calibrator. *Rplp2* was used as a reference gene. For each target, groups were compared pairwise using the Mann-Whitney test. Horizontal bars represent the median and the interquartile range. *P < 0.05; **P < 0.01.

The expression patterns of selected inflammation-related genes were validated by RT-qPCR (**Figure 5**). PM led to an increase in the mRNA levels of all examined genes, namely *Il1b*, *Il6*, *Il10*, *Tnf*, and *Ccl2*, irrespective of the feeding regimen, when compared to the sham-infected groups (*P* < 0.01, U = 0 for *Il1b*, *Il6*, *Tnf* and *Ccl2*; *P* < 0.05, U = 0 for *Il10*). Infected overfed animals showed elevated expression of *Il6* (*P* < 0.05, U = 4) and *Il10* (*P* < 0.01, U = 0) compared to the infected normal-fed group. Additionally, overfed sham-infected animals showed a slight but statistically significant increase in *Il10* levels compared to the sham-infected normal-fed rats (*P* < 0.05, U = 0).

## 4 Discussion

Evidence from rodent models implicates early life overfeeding in lasting alterations in immune function within the CNS, likely mediated by changes in microglia (18). Based on this previous evidence, it was hypothesized that infant rats overfed with maternal milk since birth would exhibit changes in their innate immune response, potentially exacerbating the loss of neurons and progenitor cells in the HC associated with PM. Unexpectedly, the neonatally overfed animals showed a significant reduction in PM-induced apoptosis in the cells of the granular and subgranular layers of the hippocampal DG (**Figure 2**). These findings were accompanied by notable alterations in the hippocampal transcriptional profile, indicating a form of neuroprotection. For instance, the *Programmed Cell Death* pathway was activated in the normally fed infected animals but not in the overfed infected animals (**Figure 4** and **Supplementary Table 2**).

Previous research has demonstrated hippocampal microgliosis in the CA1 region of infant rats following 14 days of neonatal overfeeding but not in the DG (18). Accordingly, in the current study, microglia numbers and morphology in the DG (**Figures 3B, C**) did not differ between overfed and normal-fed groups in the absence of meningitis, indicating no evidence of microgliosis in the DG after 12 days of overfeeding. However, in response to the infection, overfed rats expressed less *Aif1* in their HC (**Figure 3**), and the microglial cells in their DG presented a more branched phenotype compared to the normal-fed infected rats (**Figure 3**). Thus, overfed infant rats with PM exhibit milder microglial response when compared to the normal-fed infected animals. Consistent with these findings, the functional enrichment analysis of the hippocampal transcriptome (**Figure 4** and **Supplementary Table 2**) revealed that the *Antiviral mechanism mediated by IFN-stimulated genes* pathway is activated in the normal-fed infected animals but not in the overfed infected ones. Additionally, the *Interferon signaling pathway* and *Interferon gamma signaling* are less activated in the overfed infected group than in the normal-fed infected group. Type II interferon primes microglia [reviewed in (5):] (6, 7), and this priming is commonly thought to enhance the reactivity of microglia to subsequent immunogenic signals (30–32). Indeed, IFN-γ is a pivotal regulator of the host immune response in PM (29). Therefore, it is reasonable to hypothesize that the less pronounced microglial response observed in overfed rats during PM is likely related to the reduced activation of interferon signaling pathways, which might contribute to preventing microglial priming.

Further support for the hypothesis that overfed infant rats experience less severe CNS inflammation during PM is provided by the milder activation of pathways associated with Toll-like receptor cascades, interleukins, and chemokines signaling, as well as *Neutrophil degranulation*, in the infected overfed rats compared to the infected normal-fed rats (**Figure 4** and **Supplementary Table 2**). The attenuation of pro-inflammatory biomarkers, which are critical in the pathophysiology of PM, in infected overfed animals also supports this hypothesis (**Figure 4**). Specifically, *Mmp-3* is transcriptionally upregulated in the hippocampus of infant rats with PM, and its proteolytic activity on the extracellular matrix and its ability to increase soluble Tumor necrose factor alpha (TNF-a) levels are associated with brain injury following infection (14, 33). Triggering receptor expressed on myeloid cells 1 (TREM-1) activation, through danger- and pathogen-associated molecular patterns (DAMPs and PAMPs), has been associated with cytokine production in bacterial meningitis (29, 34). Moreover, elevated levels of Interleukin-27 (IL-27) in early life have been shown to compromise protective immunity in a mouse model of sepsis (35). Another intriguing finding is the heightened expression of Indoleamine 2,3-dioxygenase 1 (*Ido1*) in response to infection in overfed-infected animals compared to normal-fed animals. IDO1 enzyme catabolizes L-tryptophan (L-Trp) into kynurenine (KYN), thus triggering the KYN pathway. IDO1 also stimulates the activation of aryl hydrocarbon receptors (AhR), favoring an immunosuppressive state in the inflammatory microenvironment [reviewed in (36):]. Moreover, signaling of the KYN pathway in infant rats with PM has been proposed as a neuroprotective mechanism, as it compensates for the increased demand for NAD+ caused by infection and inflammation. This mechanism potentially averts energy depletion and apoptosis in the hippocampus (37). However, it has been recently shown that patients with PM exhibit higher CSF levels of quinolinic acid (QUINA), the final metabolite of the KYN pathway, compared to those with meningococcal and enteroviral meningitis (38). QUINA is a substrate for NAD+ production *de novo* but is also neurotoxic. Consequently, increased QUINA production may contribute to the poorer neurological outcomes observed in PM. The overall impact of the KYN pathway on apoptotic cell death in the HC during PM remains a subject of ongoing debate.

Additionally, the hippocampal levels of *Il1b*, *Il6*, *Il10*, *Tnf*, and *Ccl2* were directly assessed by RT-qPCR (**Figure 5**). The results corroborate those previously reported by De Luca et al. (18), who did not find differences in the hippocampal mRNA levels of *Tnf*, *Il1b*, and *Il6* between overfed and normal-fed rats at post-natal day 14. However, in the present study, infected overfed animals showed increased transcription of the genes encoding the interleukins *Il6* and *Il10* compared to infected normal-fed animals (**Figure 5**). It is important to note that while IL-6 is traditionally considered a proinflammatory cytokine, it can also exert anti-inflammatory activity depending on the immune response context [reviewed in (39):]. Borsini et al. (40) demonstrated that IL-6, synergically with other cytokines, prevented decrease in neurogenesis and reduces IL-8 and IL-1β, *in vitro*. The concurrent increase of both interleukins in infected overfed animals suggests a potential concerted action of these cytokines contributing to the observed neuroprotective effect of neonatal overfeeding. This result is further supported by the functional enrichment analysis of the hippocampal transcriptome, which disclosed the canonical pathway of *FCGR3A-mediated IL10 synthesis* as being enriched only in the overfed infected animals (**Figure 4** and **Supplementary Table 2**). Notably, even in the absence of PM, overfed animals showed higher expression of *Il10* in their hippocampi than normal-fed animals (**Figure 5**), providing additional evidence of a priming effect of overfeeding on the resident immune cells of the HC.

Another intriguing result is the activation of the *ROS and RNS production in phagocytes* pathway in the overfed group during PM, contrasting with the normal-fed infected group (**Figure 4** and **Supplementary Table 2**). This might seem paradoxical, considering that an excess of reactive oxygen species (ROS) (like superoxide anion (O2−)) and reactive nitrogen species (RNS) (such as nitric oxide (NO)) is linked to poor outcomes in patients with PM [as reviewed in (41)]. In the present study, overfeeding had no impact on the intensity of the inflammatory infiltrate in the hippocampal fissure (**Figure 1**). It is noteworthy that during PM, the main producers of ROS and RNS are blood polymorphonuclear leukocytes and other immune cells that are attracted to the CNS (42). A limitation of the current study is the absence of an assessment of hippocampal levels of reactive species.

The contrast between the sham-infected normal-fed group with the sham-infected overfed group isolated the effect of overfeeding in the absence of infection. To isolate the effect of infection, the infected normal-fed group was compared with the sham-infected normal-fed group, and the infected overfed group was compared with the sham-infected overfed group. While contrasting infected normal-fed animals with infected overfed animals could provide valuable insights into the interaction between overfeeding and infection, the number of animals used in this study was insufficient to ensure the necessary statistical power to analyze both factors simultaneously. Furthermore, this comparison presupposes that the infection is uniform across both normally fed and overfed animals. Consistent with this premise, no statistically significant difference was observed between the groups regarding bacterial titers in the cerebrospinal fluid at 18 hours post-infection, confirming comparable infection intensities in both groups. Nevertheless, our findings unveiled changes in the expression of inflammatory mediators among the sham-infected overfed animals compared to the sham-infected normal-fed group. It is conceivable that these differences could impact the trajectory of the infection beyond mere bacterial growth.

Studies have shown that changes in maternal care and nursing patterns during early life can significantly affect offspring gene expression and hypothalamic-pituitary-adrenal (HPA) axis reactivity, thereby modulating stress responses and inflammatory pathways (43–46). It is possible that the increased maternal care in the overfed group (fewer pups per dam) could have influenced the observed anti-inflammatory responses during PM. This factor was not examined in the present study. Besides, while the present study suggests potential benefits of neonatal overfeeding in the PM-induced neuroinflammation in infant rats, it is important to acknowledge its complex long-term implications. Previous studies have documented the harmful impact of neonatal overfeeding on hippocampal function in adulthood [reviewed in (17, 47, 48):]. Moreover, the long-term effects of early-life overfeeding on hippocampal function in PM survivors remain unexplored. Therefore, further research is essential to understand the temporal dynamics and mechanisms by which neonatal nutritional status interacts with PM-induced neuroinflammation across various life stages, and its long-term consequences on hippocampal function.

In conclusion, this study underscores the intricate interplay between early-life overfeeding, immune response, and neuroprotection in infant rats with PM. Contrary to expectations, neonatally overfed animals displayed a notable decrease in PM-induced apoptosis in the hippocampal DG, suggesting a potential neuroprotective impact of early-life overfeeding, potentially associated with altered microglial function.

## Data Availability

The datasets presented in this study can be found in online repositories. The names of the repository/repositories and accession number(s) can be found below: https://www.ncbi.nlm.nih.gov/, PRJNA1097644.
